# Effects of quetiapine on cognitive functioning in schizophrenia: evidence for the remyelination hypothesis?

**DOI:** 10.1007/s00406-026-02309-8

**Published:** 2026-07-04

**Authors:** Marcus Ising, Florian Raabe, Laura Fischer, Andrea Schmitt, Philipp Sämann, Kristina Adorjan, Ion-George Anghelescu, Volker Arolt, Bernhard T. Baune, Udo Dannlowski, Detlef E. Dietrich, Andreas J. Fallgatter, Christian Figge, Maria Heilbronner, Markus Jäger, Georg Juckel, Carsten Konrad, Alba Navarro-Flores, Mojtaba Oraki Kohshour, Daniela Reich-Erkelenz, Jens Reimer, Eva Z. Reininghaus, Max Schmauß, Eva C. Schulte, Fanny Senner, Carsten Spitzer, Jens Wiltfang, Jörg Zimmermann, Thomas G. Schulze, Urs Heilbronner, Monika Budde, Sergi Papiol, Peter Falkai

**Affiliations:** 1 Max Planck Institute of Psychiatry, Kraepelinstr. 2, 80804 Munich, Germany; 2 Department of Psychiatry and Psychotherapy, LMU University Hospital, LMU Munich, 80336 Munich, Germany; 3 Laboratory of Neuroscience (LIM27), Institute of Psychiatry, University of Sao Paulo, São Paulo, 05453-010 SP Brazil; 4 German Center for Mental Health (DZPG), Partner site Munich-Augsburg, Munich, Germany; 5 Institute of Psychiatric Phenomics and Genomics (IPPG), LMU University Hospital, LMU Munich, 80336 Munich, Germany; 6 University Hospital of Psychiatry and Psychotherapy, University of Bern, Bern, Switzerland; 7 Department of Psychiatry and Psychotherapy, Mental Health Institute Berlin, 14050 Berlin, Germany; 8 Institute for Translational Psychiatry, University of Münster, 48149 Münster, Germany; 9 Department of Psychiatry, University of Münster, 48149 Münster, Germany; 10 Department of Psychiatry, Melbourne Medical School, The University of Melbourne, Melbourne, Australia; 11 The Florey Institute of Neuroscience and Mental Health, The University of Melbourne, Parkville, VIC Australia; 12 Department of Psychiatry, Medical School, University Medical Center OWL, Protestant Hospital of the Bethel Foundation, Bielefeld University, Bielefeld, Germany; 13 German Center for Mental Health (DZPG), Site Jena Magdeburg Halle, Jena, Germany; 14 Center for Intervention and Research on Adaptive and Maladaptive Brain, Circuits Underlying Mental Health (C-I-R-C), Site Jena Magdeburg Halle, Jena, Germany; 15 AMEOS Clinical Center Hildesheim, 31135 Hildesheim, Germany; 16 Center for Systems Neuroscience (ZSN), 30559 Hannover, Germany; 17 Department of Psychiatry, Medical School of Hannover, 30625 Hannover, Germany; 18 Department of Psychiatry and Psychotherapy, Tübingen Center for Mental Health (TüCMH), University of Tübingen, 72076 Tübingen, Germany; 19 German Center for Mental Health (DZPG), Partner Site Tübingen, 72076 Tübingen, Germany; 20 Karl-Jaspers Clinic, European Medical School Oldenburg-Groningen, 26160 Oldenburg, Germany; 21 Department of Psychiatry II, Ulm University, Bezirkskrankenhaus Günzburg, 89312 Günzburg, Germany; 22 Department of Psychiatry, Ruhr University Bochum, LWL University Hospital, 44791 Bochum, Germany; 23 Department of Psychiatry and Psychotherapy, Agaplesion Diakonieklinikum, 27356 Rotenburg, Germany; 24 International Max Planck Research School for Translational Psychiatry (IMPRS-TP), Munich, Germany; 25 Department of Immunology, Faculty of Medicine, Ahvaz Jundishapur University of Medical Sciences, Ahvaz, Iran; 26 Department of Psychiatry and Psychotherapy, University Medical Center Hamburg-Eppendorf, 20246 Hamburg, Germany; 27 Center for Psychosocial Medicine, Academic Teaching Hospital Itzehoe, Itzehoe, Germany; 28 Division of Psychiatry and Psychotherapeutic Medicine, Research Unit for Bipolar Affective Disorder, Medical University of Graz, Graz, 8036 Austria; 29 Clinic for Psychiatry, Psychotherapy and Psychosomatics, Medical Faculty, Augsburg University, Bezirkskrankenhaus Augsburg, 86156 Augsburg, Germany; 30 Department of Psychiatry and Psychotherapy, Faculty of Medicine, University Hospital Bonn, University of Bonn, 53127 Bonn, Germany; 31 Institute of Human Genetics, Faculty of Medicine and University Hospital Bonn, University of Bonn, Bonn, Germany; 32 Centres for Psychiatry Suedwuerttemberg, 88214 Ravensburg, Germany; 33 Department of Psychosomatic Medicine and Psychotherapy, University Medical Center Rostock, 18147 Rostock, Germany; 34 Department of Psychiatry and Psychotherapy, University Medical Center Göttingen, 37075 Göttingen, Germany; 35 German Center for Neurodegenerative Diseases (DZNE), 37075 Göttingen, Germany; 36 Neurosciences and Signaling Group, Institute of Biomedicine (iBiMED), Department of Medical Sciences, University of Aveiro, Aveiro, Portugal; 37 Psychiatrieverbund Oldenburger Land gGmbH, Karl-Jaspers-Klinik, 26160 Bad Zwischenahn, Germany; 38 Department of Psychiatry and Behavioral Sciences, SUNY Upstate Medical University, Syracuse, NY USA; 39 Department of Psychiatry and Behavioral Sciences, Johns Hopkins University School of Medicine, Baltimore, MD USA

**Keywords:** Working memory, Subcortical brain volumes, White matter integrity, Cell-type specific polygenic risk

## Abstract

Postmortem findings, neuroimaging data, and in-vitro models suggest a decrease in number and density of oligodendrocytes is driving cognitive deficits in schizophrenia (SCZ). Second-generation antipsychotics are discussed to improve oligodendrocyte dysfunction with most conclusive evidence available for quetiapine (QET). We postulate that sustained QET treatment leads to cognitive improvement in SCZ, particularly, in tests with high demands for working memory function. We further hypothesize that these effects are moderated by polygenic factors associated with hippocampus-related brain volumes, general white matter integrity, and/or oligodendroglia-related SCZ risk. Using data of the prospective PsyCourse study, we identified 166 patients with SCZ spectrum disorder receiving QET at one or two consecutive visits plus 166 matched patients without QET. Polygenic scores were calculated for subcortical brain volumes, measures of white matter integrity, and for cell type-specific genetic SCZ risks. QET treatment was consistently associated with improved cognitive function independent of time, specifically, in tests with high, but not with low to medium working memory load. Polygenic analyses did not reveal significant moderation effects. In contrary, low genetic SCZ risk specific for genes related to human oligodendrocyte function was associated with higher cognitive performance independent from QET. While we observed improved cognitive performance under QET in high working memory tests, we did not find evidence that polygenic factors associated with hippocampus-related brain volumes, white matter integrity, or oligodendroglia-related SCZ risk moderate this association. Thus, our tentative findings do not provide evidence for the hypothesis that polygenic estimates of hippocampal remyelination capacities influence the association between QET and cognitive performance in SCZ.

**Supplementary Information:**

The online version contains supplementary material available at https://doi.org/10.1007/s00406-026-02309-8.

## Introduction

Schizophrenia (SCZ) and related spectrum disorders affecting about 1% of the population during lifetime [1] are regarded as one of the most severe disorders, which is reflected by disability ratings of acute symptoms in the Global Burden of Disease study reaching the highest scores across all conditions [2]. Defining features are delusions, hallucinations, disorganized thinking, disorganized or abnormal motor behavior, and negative symptoms, accompanied by cognitive impairment as another core feature contributing to poor functional outcomes [3]. Characteristic cognitive impairments in SCZ include deficits in working memory, learning, and executive function [4, 5]. These deficits tend to improve under antipsychotic treatment [6, 7], and can be further enhanced by concomitant aerobic exercise [8] or cognitive remediation training [9]. However, residual impairments remain, which further increase in chronic forms of the disorder and in later life [3]. These findings stimulated the conceptualization of SCZ as a cognitive disorder [10].

Neuroimaging studies in SCZ patients identified structural and functional abnormalities in diverse brain regions and circuits associated with cognitive impairment, which involve among others the prefrontal cortex, anterior cingulate cortex and the hippocampus [11–14]. Post-mortem studies in patients with SCZ and other psychotic disorders suggest a decrease in number and density of oligodendrocytes specifically in hippocampal regions [15, 16], which is supported by own preclinical work with patient-derived cell models indicating cell-autonomous oligodendroglial dysfunction in SCZ [17]. These impairments are assumed to affect axon myelination contributing to the observable cognitive deficits [18, 19]. Treatment strategies targeting oligodendrocyte dysfunction and axon myelination have been discussed as promising approaches for treating cognitive impairment in SCZ [20], with certain antipsychotics assumed to contribute to such effects [21]. Most conclusive data have been obtained for quetiapine (QET) [22], showing in-vitro oligodendrocyte differentiation [23], oligodendroglia outgrowth [24] as well as in-vivo neuroprotection [25] and remyelination [26, 27] in chemically demyelinated mouse models, which led to an amelioration of an induced working memory impairment in these models [27, 28]. These preclinical findings correspond with results of clinical trials suggesting small, but consistently positive effects of QET treatment on cognitive function in patients with SCZ [29, 30]. Due to its presumed remyelinating effects, quetiapine has also been discussed as a treatment option for Multiple Sclerosis [22, 31] with mixed outcomes so far [32].

Twin and family studies indicate that SCZ is a highly heritable disorder with genetic factors substantially contributing to the disease risk [33]; cognitive functions also show considerable heritability in general as well as specifically in SCZ [34]. Genome-wide association studies (GWAS) suggest largely independent effects on SCZ risk and on cognitive impairment in SCZ [35, 36]. Genetic factors also contribute to the individual variability in intracranial and subcortical brain volumes. Using a GWAS approach for magnetic-resonance-imaging derived brain volumes of ENIGMA (Enhancing NeuroImaging Genetics through Meta-Analysis [37]), and other large cohorts including the UK-Biobank [38], García-Marín and colleagues identified 529 genome-wide significant loci associated with intracranial and subcortical brain volumes, with polygenic scores of these loci explaining up to 35% of the variability observed in independent cohorts of different ancestries [39]. Van der Meer and colleagues [40] developed polygenic estimates for nine hippocampal subfield volumes with heritability estimates ≥ 20% by using brain scans of more than 20,000 individuals from 16 cohorts including the UK Biobank. Zhao and co-authors generated polygenic estimates for cerebral microstructure integrity using diffusion tensor imaging (DTI) data of 21 white matter tracts including data of more than 40,000 individuals from the UK Biobank and four other resources [41] showing an average heritability of 47.6%.

Papiol et al. tested the assumption that specific genetic risk factors for SCZ related to different stages of oligodendrocyte development may moderate hippocampal volume change following repeated aerobic endurance training in patients with SCZ [42]. This study selected the top 5% of genes specifically expressed (i) by oligodendrocyte precursor cells, (ii) by mature oligodendrocytes, and (iii) by radial glia-like cells according to the results of single-cell RNA-sequencing of mouse brain cells [43]. Then, they calculated cell-type specific polygenic risk scores (CTS-PRS) restricted to polymorphisms of the corresponding human homologues of the selected genes that are associated with an increased risk for SCZ in a genome-wide association study. The CTS-PRS of oligodendrocyte precursor cells and of radial glia-like cells were indeed inversely associated with the observed volume changes in hippocampal subfields after three months of endurance training [42], which was in part replicated in an independent cohort of SCZ patients [44]. These findings suggest a genetic moderation of the assumed remyelination effects induced by aerobic exercise.

From these findings, we postulate that a potential remyelinating effect of sustained QET treatment in SCZ should lead to improved cognitive functioning, particularly, in those tests that require activation of hippocampal memory formation. Recent neurophysiological findings from animal [45] and patient studies [46, 47] suggest that this is the case for cognitive tests demanding a high working memory capacity for successful completion. We further assume that these effects might be moderated by genetic factors associated with subcortical brain volumes, cerebral white matter integrity, and/or cell-type specific genetic risk factors for SCZ related to oligodendrocyte gene expression. The PsyCourse study [48] comprising a large longitudinal sample of patients with schizophrenia spectrum disorder with detailed data on treatment, psychopathology, and cognitive function provides a naturalistic framework to address these research questions.

## Materials and methods

### Sample

PsyCourse is a naturalistic multicentric study of 20 clinical centers in Germany and Austria that includes longitudinal data of patients with SCZ spectrum disorder, bipolar disorder, major depression, and healthy controls. Diagnoses were ascertained using an adapted version of the Structured Clinical Interview for DSM-IV, Axis I, German version [49]. Psychopathology and cognitive function were prospectively evaluated every six months at up to four times. The study protocol was approved by the responsible ethic committees of all centers, and informed consent was obtained from all participants prior study inclusion (for further details, see [48, 50]). Version 6.0 of the PsyCourse dataset was used.

We identified 106 patients with SCZ spectrum disorder (receiving sustained QET treatment (QET (+)) at two consecutive visits, which were defined as visit 1 (V1) for the first visit under QET treatment and visit 2 (V2) for the second visit with continuous QET treatment, which were 6 months apart from each other. From the sample of patients without QET treatment (QET (-)), we selected further 106 patients with SCZ spectrum disorder having two consecutive assessments at corresponding visits, which were matched pairwise for sex and age with the QET (+) patient sample. No further patient characteristics were considered for the matching procedure to ensure a sufficiently large sample size for pairwise matching. The resulting matched groups were tested for the absence of differences in additional sociodemographic and clinical variables.

Psychopathology was evaluated with the Clinical Global Impression Scale, severity index (CGI-S [51]), the Positive and Negative Syndrome Scale (PANSS [52]), the Inventory of Depressive Symptomatology, Clinician Rating (IDS-C30 [53]), and with the Global Assessment of Function (GAF) of the Diagnostic and Statistical Manual of Mental Disorders. Antipsychotic medication dosages including QET were transformed in chlorpromazine (CPZ) equivalents using a summary table (kindly provided by Lars Reitz, Cand. Med, and Michael Gottschalk, MD) harmonized across the results of a series of dose equivalence finding studies [54–58].

To enhance the statistical power of the genetic moderation analyses, we extended the sample size by additionally including 60 QET (+) patients receiving QET treatment at one single visit only (plus 60 matched QET (-) patients). DNA for these analyses was extracted from blood samples with standard methods and genotyped using Illumina Infinium Global Screening Array (GSA-24, Illumina Inc, San Diego, CA). After quality control as described elsewhere [59] genotype imputation was conducted with the Michigan Imputation Server platform (https://imputationserver.sph.umich.edu) using the Haplotype Consortium reference panel [60].

### Assessment of cognitive function

Cognitive function was assessed with the Trail Making Test (TMT-A, TMT-B [61]), with Digit Span Forward (DS-Fw), Digit Span Backward (DS-Bw) and Digit Symbol Substitution Test (DSST) of the Wechsler Intelligence Test for Adults [62], and with the Verbal Learning & Memory Test (VLMT [63]). The VLMT was performed at the second visit (V2) only, while all other tests were performed at both visits, V1 and V2. Given the different degrees of complexity between the tests, we defined the TMT-A and DS-Fw as tests requiring low to medium working memory capacity (LowWM) for maintaining and retrieving temporarily stored information. The other tests additionally require certain manipulations (TMT-B and DS-Bw) or consolidation (DSST and VLMT) of working memory information assumed to result in higher demands on working memory capacity. Thus, we defined these tests as high working memory tests (HighWM).

### Polygenic proxy scores for brain volumes/white matter integrity and cell-type specific polygenic risk scores for schizophrenia

In the absence of neuroimaging data, we calculated polygenic proxy scores for subcortical brain volumes (BV-PPS) and for DTI measures of general white matter integrity (DTI-PPS) obtained across a series of large-scale genetic neuroimaging studies [39–41]. BV-PPS scores include proxies for the volumes of the brainstem, ventral diencephalon, thalamus, whole hippocampus, hippocampal subfields (restricted to those with heritability estimates ≥ 20%: CA1, CA3, CA4, dentate gyrus, hippocampal fissure, hippocampal tail, molecular layer, presubiculum, subiculum, each corrected for whole hippocampal volume), amygdala, nucleus accumbens, caudate, putamen, globus pallidus, and for the total intracranial volume. DTI-PPS scores were calculated using five DTI parameters (fractional anisotropy, axial, radial, and mean diffusivity, mode of anisotropy) of the global average across 21 cerebral white matter tracts (for details see [41]) showing substantial heritability ranging between 50 and 65%.

In addition, we calculated conventional polygenic risk scores (PRS) for SCZ and educational attainment, using data from the most recent available genome-wide association studies [33, 64]. Cell-type specific PRS (CTS-PRS) were calculated correspondingly by identifying the top 5% of genes expressed in different cell types of the mouse brain according to the results of single-cell RNA-sequencing (RNA-seq) [43]. Scoring was restricted to polymorphisms of the corresponding human homologues of the cell type-specific genes with polygenic risk scores calculated from the results of genome-wide SCZ association studies (see [42, 44] for further details). The following CTS-PRS were included in the moderation analysis: oligodendrocyte precursor cells (OPC), mature oligodendrocytes (OLIG), radial glia-like cells, dopaminergic neurons, interneurons, and CA1 pyramidal neurons. In addition, CTS-PRS were calculated in parallel from single-nucleus RNA-seq data of corresponding human postmortem tissue [65]. Cell-type specific SCZ risk scores were again generated from the top 5% of genes expressed by human post-mortem OPC, OLIG, interneurons (originated from the medial (MGE) and caudal (CGE) ganglionic eminence), and CA1-3 hippocampal cells, with polygenic risk scores calculated from the same genome-wide SCZ association studies. Mouse and corresponding human CTS-PRS were significantly associated (after controlling for sex, age, PRS for educational attainment, and the first three ancestral principal components) with partial correlation coefficients ranging between *r* = .24 (*p* < .001, OLIG) and *r* = .42 (*p* < .001, hippocampal cells).

### Statistical analysis

Baseline QET (+) vs. QET (-) group differences in basic demographic and clinical variables were tested using Chi² tests (for categorical variables) and t-tests for independent samples (for quantitative variables). Cognitive performance was analyzed using a mixed-effects analysis of variance (ANOVA) with QET (+) vs. QET (-) as between group factor and visit 1 (V1) vs. visit 2 (V2) as within-group factor (except for the VLMT). The VLMT, which was assessed at V2 only, was analyzed with a one-way ANOVA. To limit the number of statistical tests, only the main outcome parameters were considered for analysis, which are time to finish the task (TMT-A, TMT-B), number of correct trials (DS-Fw, DS-Bw) and number of correct responses (DSST, VLMT). Normal distribution of these variables and the homogeneity of error variances between the two groups were tested with the Kolmogorov-Smirnov (KS) and the Levene tests, respectively. While KS tests of the cognitive outcome parameters indicated significant deviations from normality for the majority of variables (*p* < .001; except for DSST at both visits and VLMT at V2, with *p* = .2, each), Levene tests majorly suggested homogeneity of variance (except for TMT-A at V2, *p* = .017 and TMT-B at V1, *p* = .012). Due to the documented robustness of ANOVA tests, specifically, in case of a violation of the normality assumption [66, 67] we continued using ANOVA for these analyses, but additionally applied non-parametric Mann-Whitney U tests to ascertain potential group effects of the averaged test scores across the two visits, V1 and V2 (except for the VLMT available at V2 only). Finally, ANOVAs were repeated after including sociodemographic (age, sex, and education) and clinical variables (illness duration, psychopathology and global function at visit 1, co-treatment with typical antipsychotics, overall antipsychotic drug dosage in CPZ equivalents) as covariates to ascertain the robustness of the outcomes.

In addition, composite scores were calculated after Z-standardization (M = 100; SD = 10) of the individual test scores at V1 (except of VLMT, which was available at V2 only) assumed to reflect test results demanding low to medium (LowWM: TMT-A (-), DS-Fw) vs. high working memory capacity (HighWM: TMT-B (-), DS-Bw, DSST, VLMT). In case of missing V1 data (LowWM/HighWM: in 3.1%/4.4% of the patients), V2 data were used instead. The two composite scores did not show significant deviations from normality or homogeneity of error variances between groups (LowWM: KS test; *p* = .20; Levene test: *p* = .27; HighWM: KS test; *p* = .20; Levene test: *p* = .54). Intra-class correlation coefficients reached 0.45 for LowWM and 0.76 for HighWM, indicating acceptable reliability for the HighWM composite score.

To test for potential moderating effects of QET by BV-PPS, DTI-PPS, or CTS-PRS, we conducted an exploratory moderation analysis following an hierarchical multivariate regression approach [68, 69] to predict HighWM by applying the following steps: (1) sex, age, body height (for the BV-PPS/DTI-PPS moderation analysis only), PRS for educational attainment and the first three ancestral principal components as baseline covariates, (2) QET (+) vs. QET (-) and BV-PPS/DTI-PPS/CTS-PRS as independent predictors, and (3) the interaction term between QET (+) vs. QET (-) and the respective BV-PPS/DTI-PPS/CTS-PRS, defined as the product of the Z-standardized (M = 100; SD = 10) scores of both predictors from step (2). In case of a significant moderation of the QET effects on HighWM, we would expect significant effects for the stage (2) QET predictor as well as for the interaction term in step (3). To test for potential risks of multicollinearity within BV-PPS and CTS-PRS predictors, respectively, we calculated variation inflation factors (VIFs) for all superordinate PRS and the PRS of the subordinate subfields or cell types; tested superordinate PRS are the BV-PPS of the intracranial brain volume, the BV-PPS of the whole hippocampus volume, and the conventional SCZ PRS, with VIF < 5 defined as moderate risk and VIF < 2 as low risk for multicollinearity. The level of significance was set to *p* = .05. Analyses were performed with IBM SPSS Statistics, version 25. Given the exploratory nature of these analyses we did not correct for multiple testing; consequently, our findings should be regarded as preliminary and suggestive.

## Results

We included 212 PsyCourse patients with schizophrenia spectrum disorder, 106 of them receiving continuous QET treatment at V1 and V2 (QET (+)) and 106 patients without QET treatment (QET (-)) at both visits matched for sex and age. Basic demographic and clinical variables of both samples are presented in Table 1.

**Table 1:** Basic demographic and clinical variables of the QET (+) and QET (-) samples

	QET (+)	QET (-)	*P*
Sex	50f / 56 m	49f / 57 m	*p* = .891
Age	42.3 ± 11.8	42.8 ± 12.1	*p* = .778
Education (years)	10.6 ± 1.3	10.3 ± 1.4	*p* = .124
Diagnosis (DSM-IV)	75 × 295.3/2 × 295.4/ 29 × 295.7	88 × 295.3/1 × 295.4/ 17 × 295.7	*p* = .105
CGI-S (Visit 1)	4.07 ± 1.0	4.24 ± 1.1	*p* = .245
PANSS (Visit 1)	53.7 ± 16.2	58.3 ± 20.2	*p* = .069
IDS-C30 (Visit 1)	13.4 ± 9.6	13.8 ± 11.2	*p* = .762
GAF (Visit 1)	55.1 ± 13.6	54.0 ± 13.4	*p* = .561
Illness duration (years)	13.3 ± 9.4	14.7 ± 12.5	*p* = .375
Typical AP (yes/no)	28/78	39/67	*p* = .104
CPZ dosage	1024 ± 620	936 ± 549	*p* = .275
QET dose (mg/d)	543 ± 290	N.A.	

Group differences in sex and diagnosis were evaluated with Chi² - tests, while independent t-tests were used for all other variables

QET (+) := patients with sustained quetiapine treatment; QET (-) := patients without quetiapine treatment; CGI-S := Clinical Global Impression – Severity Scale; PANSS := Positive and Negative Symptom Scale (total score); IDS-C30 := Inventory of Depressive Symptoms, Clinician Rating; GAF := Global Assessment of Functioning scale; Typical AP := Patients (co-)treated with typical (first-generation) antipsychotics; CPZ := clozapine dose equivalents

No group differences at the level of statistically significance were observed.

**Fig. 1:**
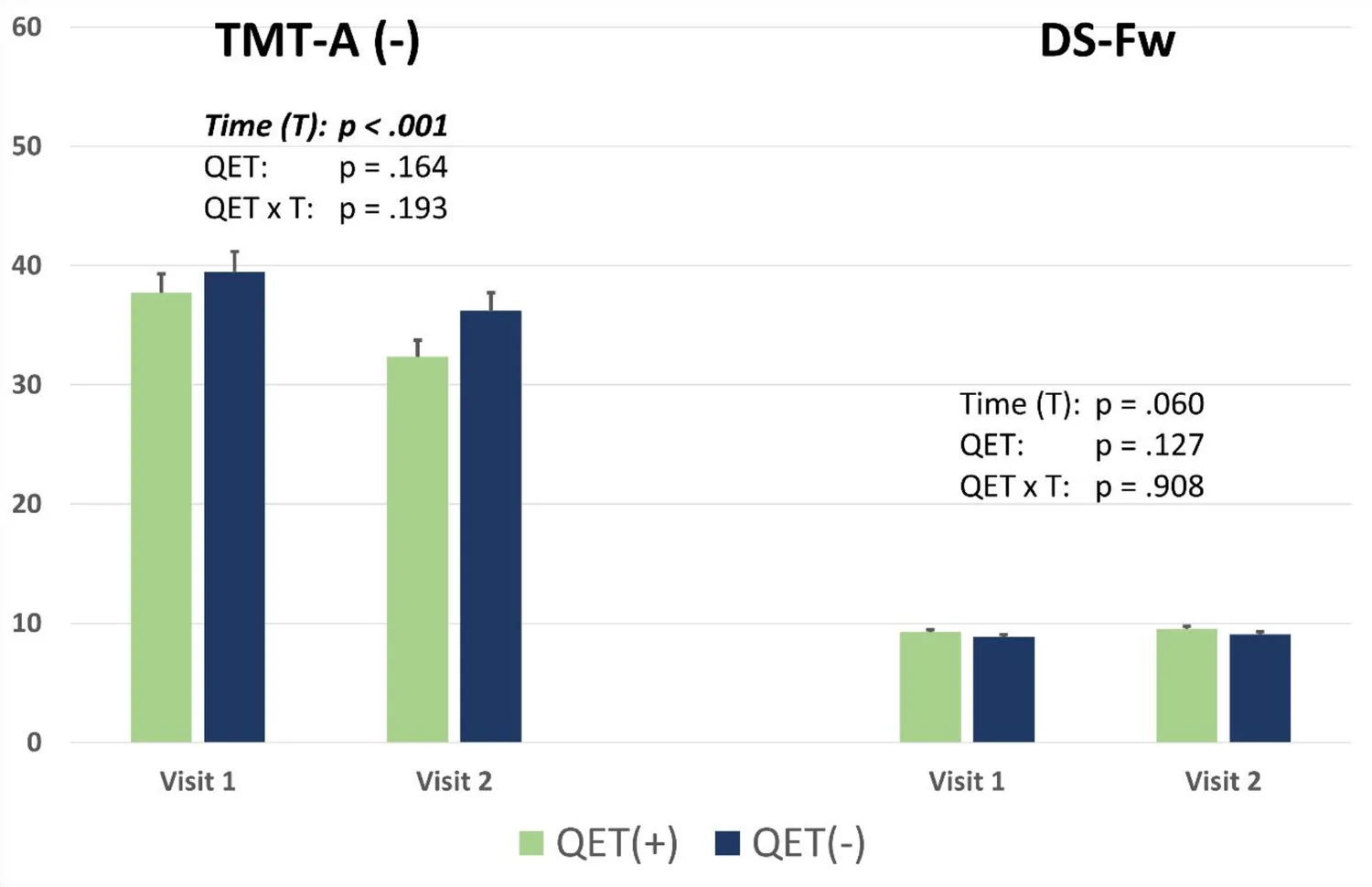
Cognitive performance in QET (+) vs. QET (-) at visits 1 and 2 in low to medium working memory tasks. Higher values reflect better performance except for the Trail Making Test, indicated by (-) (Trail Making Test A, TMT-A; Digit Span Forward, DS-Fw)

We hypothesize that the performance in tests with a high working memory load should particularly benefit from QET treatment. First, we analyzed the effects of QET treatment in low to medium working memory tasks (TMT-A, DS-Fw). Unsurprisingly, no QET main or interaction effect could be observed in the parametric ANOVA (see Fig. 1), with the absent main effect ascertained in non-parametric U tests (TMT-A V1 + V2: z = -1.58, *p* = .114; DS-Fw V1 + V2: z = -1.53, *p* = .125); the group-independent performance improved at V2 in the TMT-A (F_1,171_ = 28.92, *p* < .001) and with approaching significance also in DS-Fw (F_1,187_ = 3.29, *p* = .060).

QET main and interaction effects remained non-significant for all low to medium working memory tests after including sociodemographic (age, sex, education) and clinical variables (illness duration, psychopathology, global function, co-treatment with typical antipsychotics, overall antipsychotic drug dosage) as covariates (see Supplementary Table S1).

**Fig. 2:**
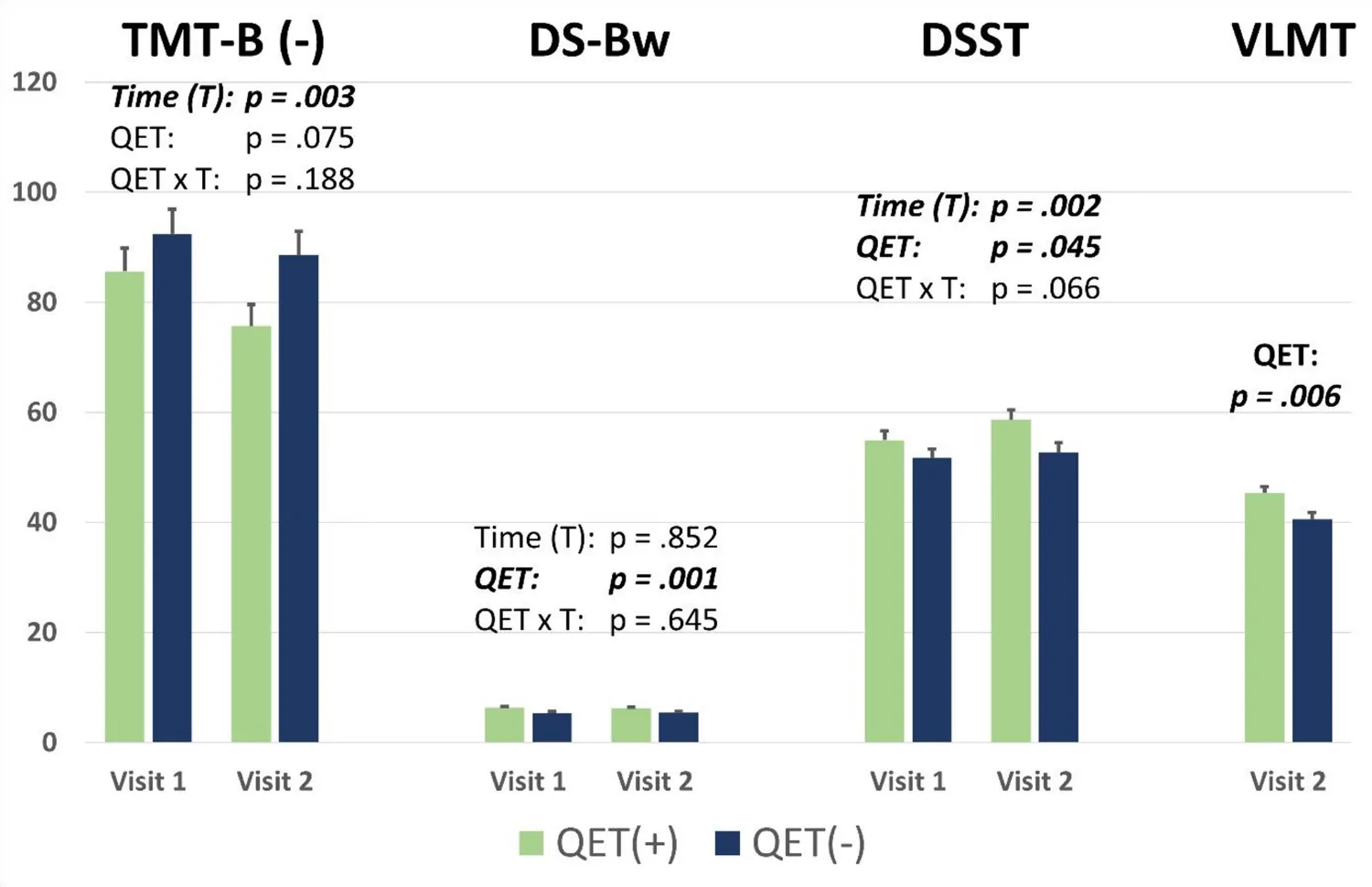
Cognitive performance in QET (+) vs. QET (-) at visits 1 and 2 (except for VLMT, which was available only for visit 2) in high demand working memory tasks. Higher values reflect better performance except for the Trail Making Test, indicated by (-) (Trail Making Test B, TMT-B; Digit Span Backward, DS-Bw; Digit Symbol Substitution Test, DSST; Verbal Learning & Memory Test, VLMT)

Next, we evaluated QET effects in tests with high working memory load including TMT-B, DSST, DS-Bw, and VLMT (see Fig. 2). In these tests, we observe significant group effects, while the interaction effects with time were non-significant, indicating a generally superior cognitive performance in QET (+) patients, except for TMT-B (F_1,171_ = 3.20, *p* = .075). When analyzing TMT-B performance at the second visit only, QET (+) patients showed significantly better performance than QET (-) also in this test (F_1,189_ = 6.67, *p* = .010). All ANOVA group effects could be successfully replicated in non-parametric U tests (TMT-B V1 + V2: z = -1.15, *p* = .249; TMT-B V2: z = -2.27, *p* = .023; DS-Bw V1 + V2: z = -3.28, *p* = .001; DSST V1 + V2: z = -2.03, *p* = .042; VLMT V2: z = -3.13, *p* = .002).

Significant QET main and non-significant interaction effects remained stable after including sociodemographic and clinical variables as covariates except for the DSST (see Supplementary Table S1), for which the significant group effect turned non-significant (F_1,178_ = 2.67, *p* = .104). However, when considering visit 2 only, a significant QET effect could be detected also for the DSST (F_1,177_ = 4.85, *p* = .029).

Given the relative uniformity of the effects across all tests with a high working memory load (TMT-B (-), DSST, DS-Bw, VLMT), we calculated a composite score (HighWM) for the respective test outcomes at visit 1 (except for VLMT, which is available only at visit 2) after Z-standardizing (M = 100, SD = 10) of the single test results. In this combined test score, QET (+) patients showed a superior performance indicated by an above average HighWM score of 101.1 (SD = 7.5), while QET (-) patients presented with a below average HighWM score of 97.8 (SD = 7.9). The group difference was highly significant (F_1,208_ = 9.42, *p* = .002) reaching an effect size of Cohen’s f = 0.21 approaching the border to a medium effect (f = 0.25) [70]. The LowWM score including the standardized results of TMT-A (-) and DS-Fw did not reach a significant QET effect (F_1,208_ = 1.81, *p* = .180).

### Effects of quetiapine dosage on HighWM

**Fig. 3:**
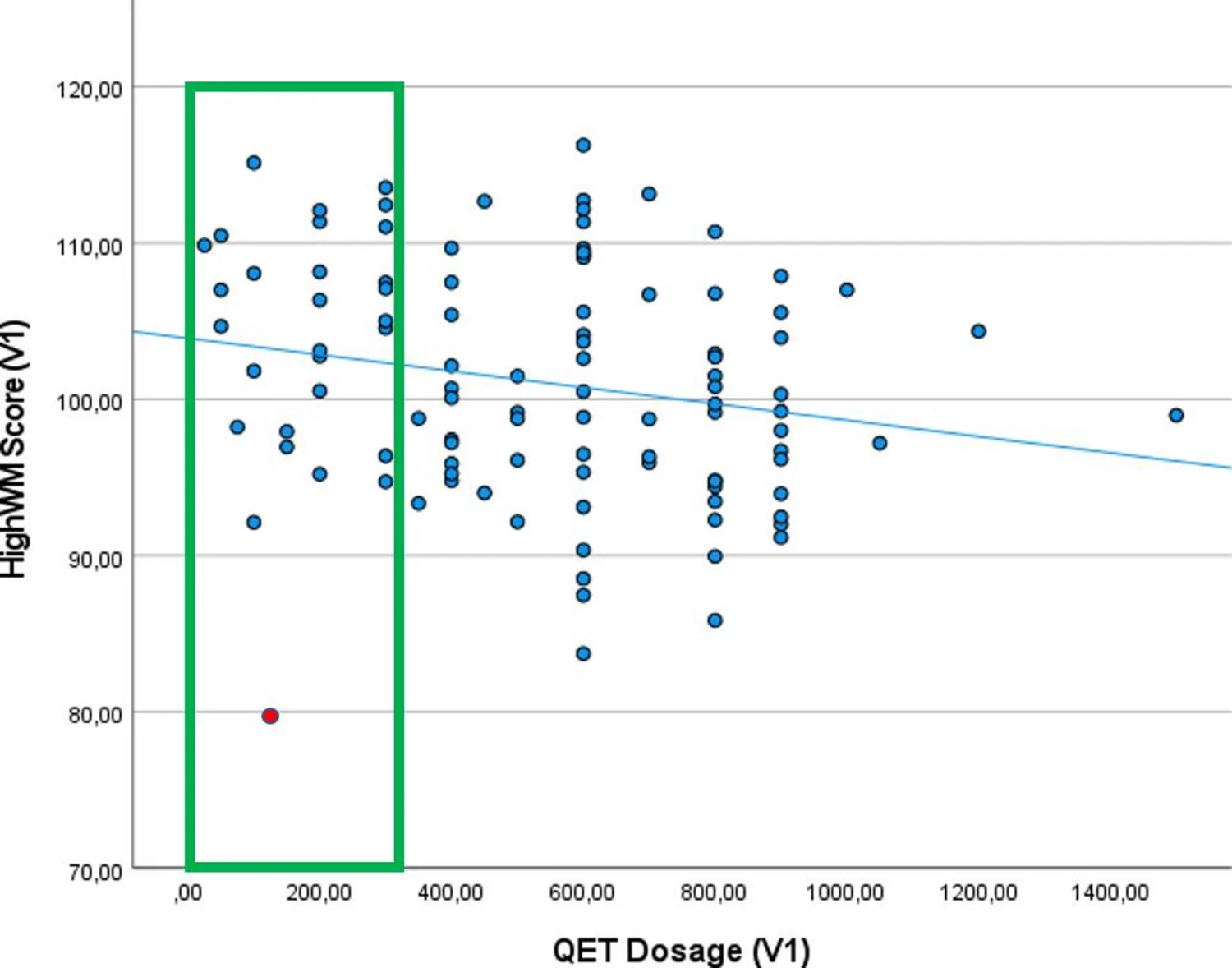
Association between QET dosage and the HighWM score at visit 1 among QET (+) patients. HighWM := Summary score of tests with high working memory demand (TMT-B (-), DS-Bw, DSST, and VLMT); QET := Quetiapine. Red dot indicates one outlier

When evaluating the association between QET dosage (at visit 1) and corresponding HighWM scores among QET (+) patients, we observe an inverse association suggesting higher performance in patients receiving lower QET dosages (ρ = − 0.23, *p* = .016; after eliminating one outlier: ρ = − 0.26, *p* = .007) with patients treated with a QET dosage of 300 mg/d or less tending to characterize a subgroup with better performance than patients with higher QET dosages (see Fig. 3).

When comparing 29 QET (+) patients receiving a daily dosage of 300 mg or less with their match partners, we could replicate the previous results. Cognitive performance in HighWM tests reached an above average score of 103.9 (SD = 7.8) in this group, while the group of QET (-) patients achieved a below average HighWM score of 97.0 (SD = 7.5). This group difference was again highly significant (F_1,52_ = 10.95, *p* = .002) with an effect size of Cohen’s f = 0.46 indicating a large effect (f > 0.40) for this group difference [70]. No differences were observed for the LowWM score (F_1,52_ = 1.22, *p* = .275).

### Identifying potential moderating effects using polygenic proxy scores for brain volumes and white matter integrity

Despite significant group effects in favor of QET (+), we also observe a considerable variability between patients suggesting the potential involvement of moderating factors, for instance, structural and functional variations in certain brain regions. In the absence of neuroimaging data for this sample, we are using polygenic proxy scores developed to estimate the variability in individual subcortical brain volumes (BV-PPS) and in general white matter integrity as reflected by DTI measures (DTI-PPS).

To enhance the power of the genetic moderation analysis and due to the fact that we see QET (+) effects already at the first visit, we extended the sample size by additionally including 60 QET (+) patients receiving QET treatment at a single visit (plus 60 matched QET (-) patients). The additional QET (+) vs. QET (-) patients did not differ in sex (*p* = 1.00), age (*p* = .943), education (*p* = .519), diagnosis (*p* = .088), CGI-S (*p* = .756), PANSS (*p* = .247), GAF (*p* = .435), illness duration (*p* = .590), number of patients (co-)treated with typical (first generation) antipsychotics (*p* = .532), or CPZ dose equivalents (*p* = .108). To test for potential moderating effects of the BV-PPS/DTI-PPS, we applied a hierarchical multivariate regression analysis for each BV-PPS/DTI-PPS to predict HighWM with sex, age, body height, the polygenic risk score for educational attainment, and the first three ancestral principal components as baseline covariates. Tested BV-PPS include proxies for the volumes of the brainstem, ventral diencephalon, thalamus, whole hippocampus, hippocampal subfields, amygdala, nucleus accumbens, caudate, putamen, globus pallidus and for the total intracranial volume. Low risks of multicollinearity were observed for the BV-PPS of the (whole) hippocampal volume (VIF = 1.05) and for the BV-PPS of the intracranial volume (VIF = 1.06). The results of the moderation analysis are presented in Table 2.

**Table 2:** Hierarchical multivariate regression to predict HighWM using QET (+) vs. QET (-) and BV-PPS as predictors

Brain volumes	Step (2a) QET (+) vs. QET (-)	Step (2b) BV-PPS	Step (3) QET x BV-PPS
Brainstem	ß = 0.151, *p* = .005	ß = − 0.093, *p* = .085	*p* = .826
Ventral diencephalon	ß = 0.157, *p* = .004	ß = − 0.074, *p* = .173	*p* = .263
Thalamus	ß = 0.156, *p* = .004	ß = − 0.087, *p* = .113	*p* = .714
Hippocampus (whole)	ß = 0.155, *p* = .004	ß = − 0.004, *p* = .936	*p* = .296
- CA1	ß = 0.157, *p* = .004	ß = 0.016, *p* = .775	*p* = .472
- CA3	ß = 0.155, *p* = .004	ß = − 0.011, *p* = .846	*p* = .604
- CA4	ß = 0.158, *p* = .004	ß = − 0.053, *p* = .331	*p* = .493
- Dentate gyrus	ß = 0.158, *p* = .003	ß = − 0.102, *p* = .063	*p* = .889
- Hippocampal fissure	ß = 0.154, *p* = .005	ß = 0.017, *p* = .751	*p* = .873
- Hippocampal tail	ß = 0.159, *p* = .004	ß = − 0.026, *p* = .637	*p* = .885
- Molecular layer	ß = 0.148, *p* = .007	ß = − 0.063, *p* = .254	*p* = .562
- Presubiculum	ß = 0.154, *p* = .005	ß = 0.038, *p* = .482	*p* = .258
- Subiculum	ß = 0.155, *p* = .005	ß = 0.003, *p* = .958	*p* = .421
Amygdala	ß = 0.156, *p* = .004	ß = − 0.025, *p* = .636	*p* = .801
Nucleus accumbens	ß = 0.155, *p* = .005	ß = 0.001, *p* = .994	*p* = .525
Caudate	ß = 0.152, *p* = .005	ß = 0.073, *p* = .178	*p* = .776
Putamen	ß = 0.158, *p* = .004	ß = − 0.034, *p* = .534	*p* = .400
Globus pallidus	ß = 0.158, *p* = .004	ß = − 0.030, *p* = .586	*p* = .972
Intracranial volume	ß = 0.155, *p* = .004	ß = 0.002, *p* = .973	*p* = .304

In Step 1 (not shown), sex, age, body height, polygenic risk score for educational attainment and the first three ancestral principal components were included as baseline covariates. Polygenic load for hippocampal subfield estimates were corrected for whole hippocampal volume in the original genome-wide association analysis [40]

HighWM := Summary score of tests with high working memory demand (TMT-B (-), DS-Bw, DSST, and VLMT); QET (+) := patients with sustained quetiapine treatment; QET (-) := patients without quetiapine treatment; BV-PPS := Polygenic proxy scores to estimate individual subcortical and intracranial brain volumes

**Table 3:** Hierarchical multivariate regression to predict HighWM using QET (+) vs. QET (-) and DTI-PPS as predictors

DTI Measures	Step (2a) QET (+) vs. QET (-)	Step (2b) DTI-PPS	Step (3) QET x DTI-PPS
Fractional Anisotropy	ß = 0.159, *p* = .003	ß = − 0.094, *p* = .083	*p* = .997
Axial Diffusivity	ß = 0.155, *p* = .004	ß = − 0.047, *p* = .385	*p* = .214
Radial Diffusivity	ß = 0.155, *p* = .004	ß = − 0.001, *p* = .984	*p* = .472
Mean Diffusivity	ß = 0.155, *p* = .004	ß = 0.029, *p* = .597	*p* = .598
Mode of Anisotropy	ß = 0.158, *p* = .004	ß = − 0.068, *p* = .210	*p* = .699

In Step 1 (not shown), sex, age, body height, polygenic risk score for educational attainment and the first three ancestral principal components were included as baseline covariates

HighWM := Summary score of tests with high working memory demand (TMT-B (-), DS-Bw, DSST, and VLMT); QET (+) := patients with sustained quetiapine treatment; QET (-) := patients without quetiapine treatment; DTI-PPS := Polygenic proxy scores to estimate individual white matter microstructure across 21 tracts [41]

DTI-PPS include fractional anisotropy, axial, radial, and mean diffusivity, and mode of anisotropy for the average integrity across 21 cerebral white matter tracts (for details see [41]); the results are presented in Table 3.

Both moderation analyses confirm consistent effects of the QET treatment condition QET (+) vs. QET (-) as significant predictor for HighWM. Beyond this, we could not detect significant associations for any of the BV-PPS or DTI-PPS suggesting no prominent role for the polygenic estimates of brain volume and general white matter integrity as independent predictors for HighWM or as moderators for the effects of QET treatment.

### Identifying potential moderating effects using cell-type specific polygenic risk scores

Further candidates for genetic moderation effects could be cell type-specific polygenic risk scores (CTS-PRS) reflecting polygenic SCZ risk based on variants of the top 5% of genes specifically expressed in different types of glial and neuronal brain cells obtained from mouse single cell RNA-seq data [42, 44]. The following CTS-PRS were considered for the moderation analysis: oligodendrocyte precursor cells (OPC), mature oligodendrocytes (OLIG), radial glia-like cells, dopaminergic neurons, interneurons, and pyramidal cells in the hippocampal Cornu Ammonis 1 (CA1) region. The conventional SCZ PRS was included as general benchmark, for which a moderate risk of multicollinearity was observed (VIF = 2.00). In addition, CTS-PRS were calculated in parallel from human single nucleus RNA-seq data for corresponding cell types [65] including OPC, OLIG, MGE and CGE interneurons as well as CA1-3 hippocampal cells. The results of the CTS-PRS moderation analysis are presented in Table 4.

**Table 4:** Hierarchical multivariate regression to predict HighWM using QET (+) vs. QET (-) and CTS-PRS as predictors

Cell types	Step (2a) QET (+) vs. QET (-)	Step (2b) CTS-PRS	Step (3) QET x CTS-PRS
Mouse OPC	ß = 0.149, *p* = .006	ß = 0.024, *p* = .666	*p* = .953
Human OPC	ß = 0.151, *p* = .005	ß = − 0.087, *p* = .108	*p* = .516
Mouse mature OLIG	ß = 0.151, *p* = .005	ß = − 0.019, *p* = .737	*p* = .853
Human OLIG	ß = 0.143, *p* = .008	ß = − 0.110, *p* = .043	*p* = .998
Mouse radial glia-like cells	ß = 0.153, *p* = .005	ß = 0.050, *p* = .358	*p* = .699
Mouse dopaminergic neurons	ß = 0.152, *p* = .005	ß = − 0.062, *p* = .248	*p* = .595
Mouse interneurons	ß = 0.151, *p* = .006	ß = 0.012, *p* = .827	*p* = .205
Human MGE interneurons	ß = 0.148, *p* = .007	ß = − 0.022, *p* = .681	*p* = .475
Human CGE interneurons	ß = 0.148, *p* = .006	ß = − 0.048, *p* = .380	*p* = .213
Mouse CA1 pyramidal cells	ß = 0.151, *p* = .005	ß = 0.017, *p* = .753	*p* = .324
Human CA1-3 cells	ß = 0.151, *p* = .005	ß = 0.017, *p* = .750	*p* = .727
Schizophrenia PRS	ß = 0.148, *p* = .006	ß = − 0.028, *p* = .607	*p* = .980

In Step 1 (not shown), sex, age, polygenic risk score for educational attainment and the first three ancestral principal components were included as baseline covariates. In addition to CTS-PRS, the conventional schizophrenia PRS was included as general benchmark

HighWM := Summary score of tests with high working memory demand (TMT-B (-), DS-Bw, DSST, and VLMT); QET (+) := patients with sustained quetiapine treatment; QET (-) := patients without quetiapine treatment; CTS-PRS := Cell-type specific polygenic risk score (PRS) for schizophrenia; OPC: oligodendrocyte precursor cells; OLIG := oligodendrocytes; MGE/CGE := medial/caudal ganglionic eminence; CA1-3 := hippocampal subfields

Once again, the moderation analysis consistently shows significant effects for the QET treatment condition QET (+) vs. QET (-) as predictor of HighWM, while no significant moderation effects could be identified for any of the CTS-PRS. In contrary, the CTS-PRS for genes expressed by oligodendrocytes according to human RNA-seq data showed a QET independent negative association with HighWM (see Table 3). This suggests that a low genetic SCZ risk related to human oligodendrocyte function contributes to higher cognitive performance independent from the actual QET treatment. The corresponding mouse CTS-PRS did not show an independent prediction effect on HighWM.

## Discussion

Using data of the prospective PsyCourse study [48], we identified 106 patients with SCZ spectrum disorder receiving QET treatment at two consecutive visits six months apart plus 106 patients without QET treatment matched for sex and age. The two groups did not differ in basic demographic and clinical parameters.

Previous findings [12, 42, 44] suggested that a potential remyelinating effect could lead to improved cognitive functioning in tests requiring activation of hippocampal memory formation. We assumed that this is the case for cognitive tests demanding a high working capacity for a successful completion [45–47]. Indeed, patients under QET treatment outperformed patients without QET in all cognitive tests of high working memory load, except for the TMT-B, which showed a suggestive group effect with approaching significance (*p* = .075) and a significant group effect only at visit 2. Group effects could be replicated after additionally controlling for sociodemographic and clinical variables with the exception of the DSST, for which a significant QET effect could be ascertained only at visit 2. In this context, it might be important to note that the large majority of patients in the QET (-) group (87%) were also treated with second-generation antipsychotics except QET across both visits, with the rate of patients (co-)treated with typical (first-generation) antipsychotics not statistically different between both treatment groups. These findings support the assumption that the observed group association with cognitive function is not a general result of an atypical antipsychotic medication, but rather specific for QET.

In addition, we could show that the association between QET and cognitive function is restricted to tests with high working memory demand (HighWM), while similar cognitive performance was observed in both treatment groups for less demanding tests (LowWM: TMT-A, DS-Fw) primarily measuring perceptual abilities, psychomotor speed, and attention [71, 72]. The HighWM tests TMT-B, DSST, DS-Bw, and VLMT majorly reflect executive and working memory function [63, 71, 73, 74] and are assumed to represent more complex network capacities including the hippocampus [75, 76]. For these tests, we observed highly significant QET effects at the border of a medium effect size (f = 0.21).

Beneficial effects of QET on cognitive function in SCZ have been reported earlier. Meta-analyses showed that treatment with second-generation antipsychotics improves cognitive function more than typical antipsychotics [29, 30]. Consistent effects across cognitive domains were found for QET compared with other second-generation antipsychotics, which appear to improve cognitive function for specific domains only [29]. A recent meta-analysis [7] restricted to randomized placebo-controlled monotherapy trials in SCZ confirmed that first-generation dopamine antagonists like haloperidol and the second-generation antipsychotic clozapine reached worse outcomes on general cognitive performance than QET, while no significant differences were found between QET and other second-generation antipsychotics. As this meta-analysis was restricted to monotherapy trials, the QET dosage was rather high reaching an average dosage across all studies of approximately 500 mg/d. Only five out of the 13 studies permitted dosages of less than 300 mg/d [7]. In this context, it is important to note that we observed the strongest association when the QET (+) group was restricted to patients treated with a QET dosage of 300 mg/d or less (vs. corresponding QET (-) match partners) reaching an effect size of a large effect (f = 0.46). Thus, the restriction to monotherapy trials with comparably high QET dosages might explain, why no beneficial effect for QET compared to other second-generation antipsychotics was observed in this meta-analysis.

Contrary to our initial expectation, the QET effect was present already at the first visit and did not tend to further increase at the second visit indicated by the absence of interaction effects between QET and visit. Detailed information about when the current treatment had actually been initiated prior to the first visit is unfortunately not available for this sample. However, the average illness duration of more than 13 years in combination with the non-interventional and observational study design suggest that the concurrent treatment of the patients at visit 1 was most likely initiated quite some time earlier.

Searching for genetic mechanisms driving the suggested QET effects, we analyzed the moderating role of polygenic proxy scores for cortical and subcortical brain volumes (BV-PPS), for cerebral white matter integrity (DTI-PPS), and of cell-type specific polygenic risk scores underlying the genetic SCZ risk (CTS-PRS) in an extended sample by additionally including 60 QET (+) patients receiving QET treatment at a single visit only (plus 60 matched QET (-) patients) (*N* = 2 × 166 patients). QET groups of the extended sample did not differ in basic demographic and clinical parameters.

We could not identify a significant moderation effect for any of the investigated BV-PPS or DTI-PPS suggesting that the observed QET effects are apparently independent of genetically determined estimates for subcortical brain volumes and for general white matter tract integrity. This could be a consequence of a generally limited predictive capacity of these polygenic estimates (see also [39, 41]), and related to the fact that the scores were not specifically developed for schizophrenia, but generated from general neuroimaging databases that include a variety of clinical and non-clinical samples.

Likewise, we did not detect a significant moderation effect for both types of CTS-PRS, derived from mouse single-cell and from human single-nucleus RNA-seq. However, the CTS-PRS specific for genes expressed by human oligodendrocytes showed a QET independent negative association with HighWM, which suggests that a low genetic SCZ risk related to human oligodendrocyte function contributes to higher cognitive performance independent from the actual QET treatment. However, this finding was not replicated for the corresponding mouse CTS-PRS.

Post-mortem studies in patients with SCZ give evidence for a decrease in number and density of oligodendrocytes specifically in hippocampal regions [15, 16] that are assumed to be responsible for cognitive deficits observed in these patients [18, 19]. A series of preclinical studies suggests that treatment with QET may have the potential to revert hippocampal demyelination to a certain degree. For instance, in-vitro studies showed oligodendrocyte differentiation [23, 26] and oligodendroglia outgrowth [24] after QET administration. In-vivo studies in mice treated orally or peritoneally with comparably low QET dosages (5 to 10 mg/kg) showed protective effect against chemical demyelination after two to four weeks of treatment [25, 26] as well as an induced remyelination [26, 27] leading to an amelioration of the cognitive impairment in these models [28].

The results of our moderation analysis do not support the assumption that polygenic estimates of hippocampal remyelination capacities influence the observed association between QET and cognitive performance. The BV-PPS analysis did not reveal a moderation effect for polygenic estimates of subcortical volumes of hippocampus-related networks, and the DTI-PPS analysis did not indicate a moderating role for genetically determined white matter integrity. Furthermore, the CTS-PRS moderation analysis could not find evidence for an involvement of polygenic SCZ risk scores specific to oligodendroglial lineage. In contrary, low genetic SCZ risk specific for genes related to human oligodendrocyte function was associated with higher cognitive performance independent from QET. This finding suggests that cognitive performance in SCZ might be affected by genetic factors related to oligodendrocyte function, but this association appears to be without relevance for the presumed effects of QET on cognitive performance.

Other polygenic pathways that could explain potential QET effects on cognitive function might be related to the modulation of neurotrophic factors and to the modulation of inflammatory pathways and immune function. For instance, QET has been shown to stimulate the expression of neurotrophic factors in the hippocampus [77, 78] and promotes hippocampal synaptic plasticity [79]. Furthermore, sustained QET treatment demonstrated the potential of suppressing pro-inflammatory and stimulating anti-inflammatory cytokines [80], which has been discussed as another pathway contributing to the clinical effects of QET [81].

In summary, we could confirm that QET treatment in SCZ spectrum disorder is associated with improved cognitive function, particularly, in complex tests with a high working memory load. These effects appeared more pronounced in patients treated with low QET dosages. Contrary to our expectation, we could not show that polygenetic factors associated with hippocampus-related brain volumes, general white matter integrity, or with oligodendroglial function moderate the observed QET effects. Thus, our findings do not provide evidence that polygenic estimates of hippocampal remyelination capacities influence the observed association between QET treatment and cognitive performance in SCZ.

Several limitations related to the naturalistic design of the study need to be mentioned. First, treatment groups were not randomized but defined according to the prescribed antipsychotic medication and matched pairwise for sex and age only, which could have introduced a certain selection bias related to the chosen medication. We cannot exclude such a bias. However, the absence of significant group differences in basic demographic and clinical parameters as well as the pattern of the effects (HighWM, but not LowWM) majorly replicated after additionally controlling for these variables suggest robustness of the reported findings. Nonetheless, other clinical factors like certain comorbidities or medication history not available for analysis could have interfered with the outcomes of this study, which together with the non-randomized design challenge a causal interpretation of the group effects. Second, no information about the time of initiation of the current treatment was available in both groups. This in combination with the average illness duration of more than 13 years suggests that the concurrent treatment at visit 1 may have been initiated much earlier in most cases. Third, in the absence of structural and functional neuroimaging and transcriptomic data we used polygenic risk and proxy scores for the moderation analyses. Both approaches are indirect, do not cover neuroplasticity, but rely on genetic information only bearing the risk of pleiotropy or genocopy. Fourth, besides QET, there are further potential interventions assumed to promote remyelination, for instance, aerobic exercise [42] or treatment with the antiallergic compound clemastine [82], which have not been addressed in this study. We also cannot determine based on our findings if the observed QET effects are indeed specific for SCZ.

The tentative character of this study needs to be taken into consideration. Thus, findings have to be regarded as preliminary requiring replication in larger cohorts and in randomized clinical trials with repeated multimodal neuroimaging including measures of hippocampal myelination and neuroplasticity to further elucidate the neurobiological effects of QET on cognitive performance.

## Supplementary Information

Below is the link to the electronic supplementary material.

Supplementary Material 1

## Data Availability

All data used in this study were obtained from the PsyCourse project (http://www.psycourse.de/index-en.html). PsyCourse is committed to Open Science, and data are made available upon acceptance of a submitted scientific research proposal, which is reviewed by a panel of experts. Data were requested with proposal no. 094, with two submitted amendments specifying the conducted analyses.
