# Supplementary material for: Effects of quetiapine on cognitive functioning in schizophrenia: evidence for the remyelination hypothesis?
Source: Eur Arch Psychiatry Clin Neurosci. 2026 Jul 4;276(7):3493–507. doi: 10.1007/s00406-026-02309-8 (PMC13562149; doi:10.1007/s00406-026-02309-8)
Supplement: Supplementary file 1 [file 406_2026_2309_MOESM1_ESM.docx]

Supplementary Table S1: Results of the complementary analysis of covariance on the cognitive performance in QET (+) vs. QET (-) at visits 1 and 2 (time) after including sociodemographic and clinical variables as covariates.

|  | **ANCOVA Effects** | | |
| --- | --- | --- | --- |
| **Cognitive Test** | **Time** | **QET** | **QET x Time** |
| **TMT-A** | F_1,161_ = .64, p = .425 | F_1,161_ = 2.33, p = .129 | F_1,161_ = 1.40, p = .239 |
| **DS-Fw** | F_1,177_ = .11, p = .744 | F_1,177_ = 1.61, p = .206 | F_1,177_ = .01, p = .994 |
| **LowWM** |  | F_1,197_ = 1.35, p = .247 |  |
| **TMT-B** | F_1,161_ = 1.00, p = .319 | **F_1,161_ = 4.39, p = .038** | F_1,161_ = 1.81, p = .181 |
| **DS-Bw** | F_1,177_ = .01, p = .952 | **F_1,177_ = 7.16, p = .008** | F_1,177_ = .03, p = .859 |
| **DSST** | F_1,178_ = 1.41, p = .237 | F_1,178_ = 2.68, p = .104^1^ | F_1,178_ = 3.71, p = .056 |
| **VLMT** |  | **F_1,170_ = 8.56, p = .004** |  |
| **HighWM** |  | **F_1,193_ = 8.09, p = .005** |  |

*Note*. Included covariates are age, sex, education, illness duration, psychopathology and global function at visit 1, co-treatment with typical antipsychotics, overall antipsychotic drug dosage in CPZ equivalents.
^1^When considering visit 2 only, a significant QET group effect could be detected also for the DSST (**F_1,177_ = 4.85, p = .029**).
QET (+) := patients with sustained quetiapine treatment; QET (-) := patients without quetiapine treatment; TMT-A: Trail Making Test A; DS-Fw: Digit Span Forward; LowWM: standardized summary score of TMT-A (-) and DS-Fw; TMT-B: Trail Making Test B; DS-Bw: Digit Span Backward; DSST: Digit Symbol Substitution Test; VLMT: Verbal Learning & Memory Test; HighWM := standardized summary score of TMT-B (-), DS-Bw, DSST, and VLMT.
